# LV Dyssynchrony Is Helpful in Predicting Ventricular Arrhythmia in Ischemic Cardiomyopathy After Cardiac Resynchronization Therapy: A Preliminary Study

**DOI:** 10.1097/MD.0000000000002840

**Published:** 2016-02-18

**Authors:** Shih-Chuan Tsai, Yu-Cheng Chang, Kuo-Feng Chiang, Wan-Yu Lin, Jin-Long Huang, Guang-Uei Hung, Chia-Hung Kao, Ji Chen

**Affiliations:** From the Department of Nuclear Medicine (S-CT, W-YL) and Cadiovascular Center (Y-CC, K-FC, J-LH), Taichung Veterans General Hospital; Department of Medical Imaging and Radiological Sciences (S-CT, G-UH), Central Taiwan University of Science and Technology; Department of Nuclear Medicine (G-UH), Chang Bing Show Chwan Memorial Hospital, Changhua, Taiwan; Department of Medical Imaging and Radiological Sciences, China Medical University, Taichung, Taiwan (G-UH, C-HK); Department of Medicine, School of Medicine, Institute of Clinical Medicine, and Cardiovascular Research Institute, National Yang-Ming University, Taipei, Taiwan (J-LH); and Department of Radiology and Imaging Sciences (JC), Emory University School of Medicine, Atlanta, GA.

## Abstract

For patients with coronary artery disease, larger scar burdens are associated with higher risk of ventricular arrhythmia. Left ventricular (LV) dyssynchrony is associated with increased risk of sudden cardiac death in patients with heart failure. The purpose of this study was to assess the values of LV dyssynchrony and myocardial scar assessed by myocardial perfusion SPECT (MPS) in predicting the development of ventricular arrhythmia in ischemic cardiomyopathy.

Twenty-two patients (16 males, mean age: 66 ± 13) with irreversible ischemic cardiomyopathy received cardiac resynchronization therapy (CRT) for at least 12 months were enrolled for MPS. Quantitative parameters, including LV dyssynchrony with phase standard deviation (phase SD) and bandwidth, left ventricular ejection fraction (LVEF), and scar (% of total areas), were generated by Emory Cardiac Toolbox. Ventricular tachycardia (VT) and ventricular fibrillation (VF) recorded in the CRT device during follow-up were used as the reference standard of diagnosing ventricular arrhythmia. Stepwise logistic regression analysis was performed for determining the independent predictors of VT/VF and receiver operating characteristic (ROC) curve analysis was used for generating the optimal cut-off values for predicting VT/VF.

Nine (41%) of the 22 patients developed VT/VF during the follow-up periods. Patients with VT/VF had significantly lower LVEF, larger scar, larger phase SD, and larger bandwidth (all *P* < 0.05). Logistic regression analysis showed LVEF and bandwidth were independent predictors of VT/VF. ROC curve analysis showed the areas under the curves were 0.71 and 0.83 for LVEF and bandwidth, respectively. The optimal cut-off values were <36% and > 139° for LVEF and bandwidth, respectively. The sensitivity, specificity, positive predictive value, and negative predictive value were 100%, 39%, 53%, and 100%, respectively, for LVEF; and were 78%, 92%, 88%, and 86%, respectively, for bandwidth.

LV dyssynchrony as assessed by phase analysis of MPS is helpful for predicting ventricular arrhythmia in ischemic cardiomyopathy after CRT. Further implantation of defibrillator may be considered for those patients with bandwidth >139°.

## INTRODUCTION

For patients with ischemic cardiomyopathy and nonresponsive to revascularization and medical therapy, cardiac resynchronization therapy (CRT) has been proved to be effectively reduce the mortality and improve the quality of life.^1,2^ However, sudden cardiac death (SCD) secondary to ventricular arrhythmia is still the major threat for these patients.^3,4^ For reducing SCDs in heart failure (HF), implantable cardioverter defibrillator (ICD) was found to be better than anti-arrhythmic medications.^5^ Similarly, CRT combined defibrillator (CRT-D) was also better than CRT pacemaker alone in reducing the mortality.^6^ Given with high expense of the procedure, however, it is important to identify which patients should be implanted with CRT-D.

As a noninvasive cardiac imaging modality, myocardial perfusion SPECT (MPS) provides 1-stop-shop assessments of myocardial perfusion, viability, left ventricular (LV) volumes, ejection fraction (EF), and systolic/diastolic dyssynchrony.^7,8^ Using an invasive technique of electrophysiological stimulation, Gradel et al^9^ showed that myocardial scar as assessed by MPS was significantly correlated with inducible ventricular tachycardia (VT). In recent, the LV dyssynchrony parameters as quantitated by phase analysis of MPS was also found to be independent predictors of appropriate ICD shocks and SCD events.^10,11^ Using the ventricular arrhythmic data, including VT and ventricular fibrillation (VF), recorded in the CRT pacemakers as reference standard, this study was aimed to evaluate the predictive values of LV dyssynchrony, EF, and scar burden as assessed by MPS in patients with ischemic cardiomyopathy after CRT.

## MATERIALS AND METHODS

### Patients

From January 2012 to December 2014, 22 patients who had ischemic cardiomyopathy confirmed by coronary angiography and received CRT implantation in Taichung Veterans General Hospital were enrolled in this study. All the patients received CRT matched the following indications: HF with severe symptoms with New York Heart Association (NYHA) class III or IV but not responsive to invasive revascularizations and/or optimal medical treatments; left bundle branch block on baseline electrocardiogram (ECG) with a wide QRS complex (more than 120 ms) and rS or QS morphology at V1 and V2 leads; left ventricular ejection fraction (LVEF) <35% on 2-dimensional echocardiography with LV end-diastolic diameter larger than 55 mm. The patients with atrial fibrillation or significant comorbidity with short life expectancy were excluded from this study. The study protocol was approved by the Institutional Review Board of Taichung Veterans General Hospital. All enrolled patients had signed the informed consent forms.

After implantation of CRT, the patients received regular follow-up in cardiovascular clinic, and the CRT devices were interrogated at each visit. All of the CRT pacemakers permitted full disclosure of arrhythmia. VT was defined as ventricular tachyarrhythmia with regular cycle length 320 to 400 ms with or without VA dissociation. VF was defined as ventricular tachyarrhythmia with cycle length <320 ms with irregularity leading to syncope or ICD therapy.^12^ Episodes of VT and/or VF detected by the implanted device were validated by 2 electrophysiologists.

### Imaging Protocol and Analysis

All patients were referred for a resting protocol of MPS after at least 12 months of CRT. Under bi-ventricular pacing, ECG-gated MPS was performed using a dual-head SPECT camera (BrightView, Philips Healthcare, Cleveland, Ohio) 1 h after intravenous injection of 20 mCi of ^99m^Tc-sestamibi. The images were acquired with a step-and-shoot acquisition, 25 s per stop, 32 stops over the 180° orbit, 64 × 64 matrix with 6.4 mm per pixel, and 8-bin gating. The images were reconstructed using standard iterative reconstruction (ordered subsets expectation maximization with 3 iterations and 8 subsets) and Butterworth filtering (cut-off frequency 0.4 cm per cycle and power of 10).

After reconstruction, Emory Cardiac Toolbox with phase analysis was used for generating quantitative parameters, including LVEF, myocardial scar (total areas with myocardial activity < 50% of maximal normalized activity on polar map), phase standard deviation (phase SD), and bandwidth as the previously used protocol.^13^

### Statistical Analysis

For the patient characteristics, noncontinuous variables (number and percentage) were tested with Chi-squared test and continuous variables (mean ± SD) were tested with Student *t* test. Stepwise logistic regression was performed for determining the independent predictors of VT/VF and receiver operating characteristic (ROC) curve analysis was used for generating the optimal cut-off values for predicting VT/VF. A *p* < 0.05 was considered statistically significant.

## RESULTS

**FIGURE 1:**
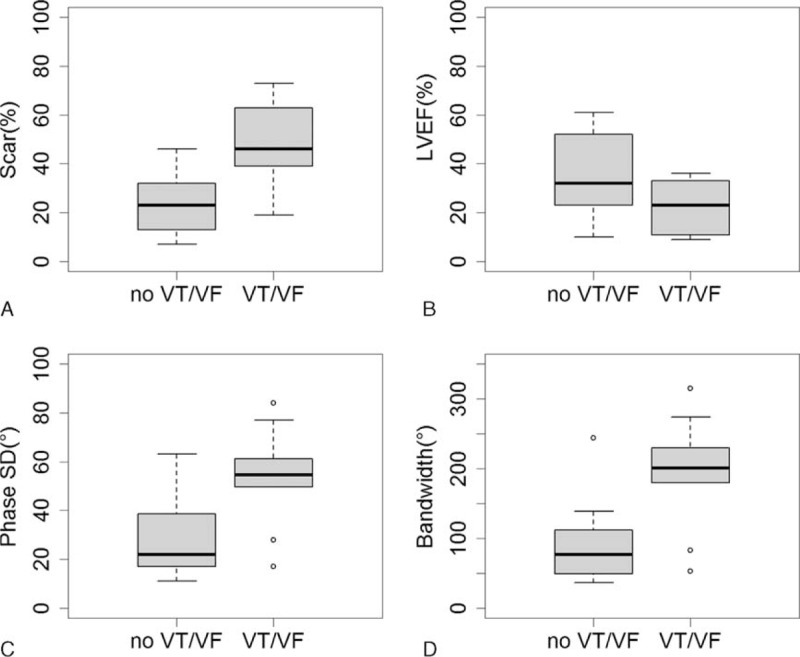
Box-and-whisker plot of myocardial scar, LVEF, phase standard deviation (phase SD), and bandwidth in all patients with (VT/VF) and without ventricular arrhythmia (no VT/VF). LVEF = left ventricular ejection fraction, VF = ventricular fibrillation, VT = ventricular tachycardia.

During the periods of follow-up (15.3 ± 12.7 months), 9 (41%) of the 22 patients developed VT/VF (6 VTs and 3 VFs). Table 1 shows the clinical characteristics and quantitative MPS parameters of the enrolled patients with and without VT/VF. Except for less hypertension for patients with VT/VF, no significant difference was noted in age, gender, body mass index, NYHA class, diabetes, or creatinine level between the patients with and without VT/VF. With regard to the quantitative parameters as assessed by MPS, the patients with VT/VF had significantly lower LVEF, larger scar, larger phase SD, and larger bandwidth. Figure 1 shows the box-and-whisker plot of myocardial scar, LVEF, phase SD, and bandwidth in all patients with and without VT/VF.

**TABLE 1:**
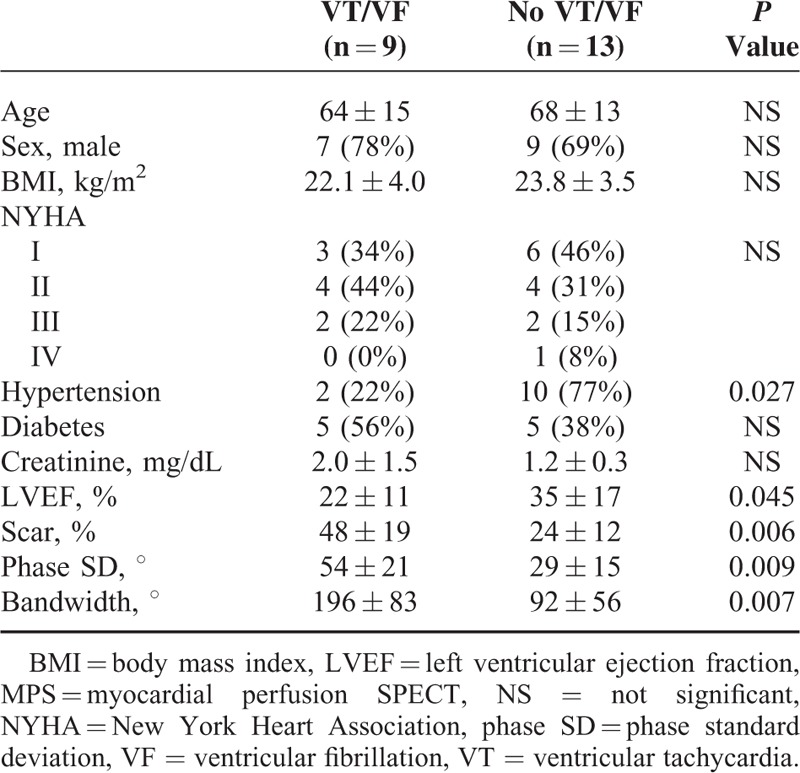
Clinical Characteristics and Quantitative MPS Parameters of the Enrolled Patients With and Without VT/VF

**FIGURE 2:**
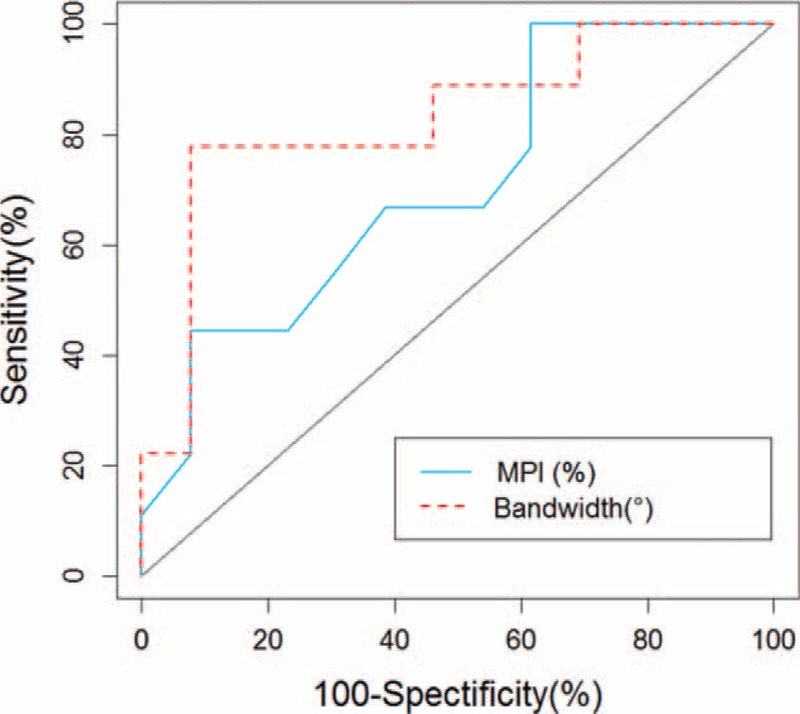
Receiver operating characteristic (ROC) curves of LVEF and bandwidth for predicting the development of ventricular arrhythmia. LVEF = left ventricular ejection fraction.

Table 2 shows the result of stepwise logistic regression analysis of the quantitative MPS parameters for predicting the development of VT/VF. LVEF and bandwidth were independent predictors of VT/VF. ROC curve analysis showed the areas under the curves were 0.71 and 0.83 for LVEF and bandwidth, respectively (Figure 2). The optimal cut-off values were <36% and >139° for LVEF and bandwidth, respectively. The sensitivity, specificity, positive predictive value, and negative predictive value were 100%, 39%, 53%, and 100%, respectively, for LVEF; and were 78%, 92%, 88%, and 86%, respectively, for bandwidth.

**TABLE 2:**
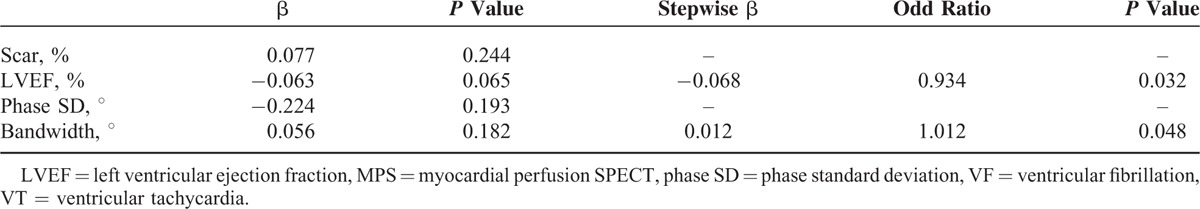
Stepwise Logistic Regression Analysis of the Quantitative MPS Parameters for Predicting the Development of VT/VF

**FIGURE 3:**
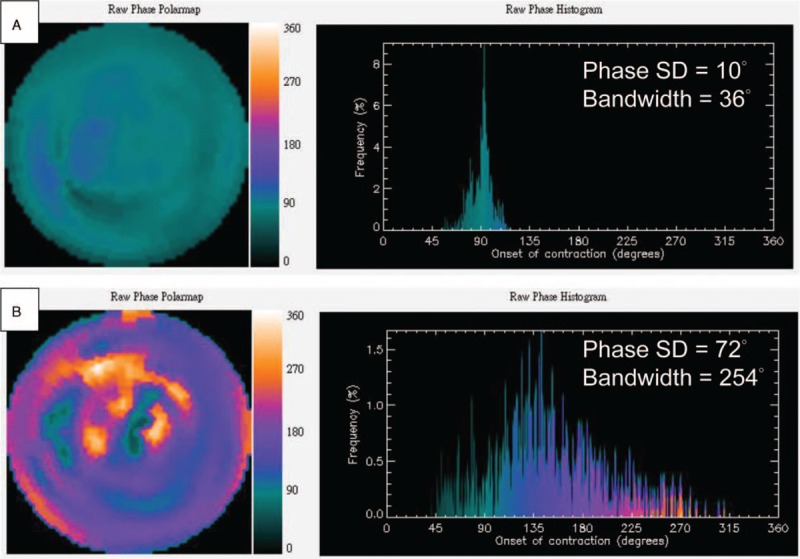
Example images from ischemic cardiomyopathy patients with cardiac resynchronization therapy who were found to have no episode (A) and have episode (B) of ventricular arrhythmia.

Figure 3 shows example images from ischemic cardiomyopathy patients with CRT. The first one was an 82-year-old female (Figure 3A) whose phase analysis of MPS showed synchronous mechanical activation with a phase SD of 10° and bandwidth of 36°. She was not found to have any episode of ventricular arrhythmia (VT/VF) during the period of follow-up. The other was a 75-year-old male (Figure 3B) whose phase analysis of MPS showed remarkably dyssynchronous activation with a phase SD of 72° and bandwidth of 254°. He was found to have episodes of VT during follow-up.

## DISCUSSION

The main finding of this study was that LV dyssynchrony as assessed by phase analysis of MPS was helpful for predicting the development of ventricular arrhythmia (VT/VF) for the ischemic cardiomyopathy patients with CRT. During the periods of follow-up, the incidence of VT/VF was as high as 41%. LV dyssynchrony parameter with bandwidth >139° provided satisfied accuracy in the diagnosis of VT/VF. This finding implied the potential role of LV dyssynchrony by phase analysis in selecting CRT patients for further revising their device as CRT-D.

With regard to the other independent predictor of VT/VF in our study, LVEF (<36%) was found to be a very sensitive predictor for VT/VF. However, its specificity was as low as only 39%. This result was consistent with the current clinical experience that implanting ICD in patients with LVEF < 35% did significantly reduce the mortality related to fatal arrhythmia; however, the average annual rate of appropriate ICD shocks was only 5.1%.^14^

In the study of Gradel et al, they investigated the relationship of myocardial scar as assessed by MPS and the development of ventricular arrhythmia. It was shown that inducible VT on electrophysiological stimulation was significantly related to the extent of myocardial scar.^9^ The underlying pathophysiologic mechanism of developing ventricular arrhythmia had been believed that myocardial scar was the anatomic substrate for reentry.^15^

In addition to LVEF and myocardial scar, the images of MPS can also be used to evaluate LV dyssynchrony which was expressed by phase SD and bandwidth by using the technique of phase analysis.^16^ All these information were very useful for guiding CRT for selecting patients with LV dyssynchrony, implanting LV lead at latest activation site and avoiding the scar area.^17,18^ In recent, LV dyssynchrony by phase analysis was found to have prognostic value for HF patients. In patients received ICD, Aljaroudi et al^10^ found that LV dyssynchrony was predictive of cardiovascular events. In their study, the patients with events had significantly larger phase SD than those without events regarding all-cause death or appropriate ICD shocks. Besides, the study showed that phase SD < 50° was associated with no events within 1 year. In the present study, we also found a similar result that LV dyssynchrony is a useful marker for the development of ventricular arrhythmia which was detected by pacemaker of CRT device. In recent, Hage et al^11^ studied the relationship of LV dyssynchrony and SCD events in HF patients. They found that patients who experienced SCD events had significantly larger phase SD than matched control patients and provided incremental prognostic information than current indicators of SCD risks.

The major limitation of our study was that the patient population was small. It was because that the HF patients referred for CRT were mainly secondary to nonischemic dilated cardiomyopathy and the patients with ischemic cardiomyopathy is relative rare. However, the value of LV dyssynchrony as assessed by MPS should be further validated in studies with larger populations.

## CONCLUSIONS

According to the ventricular arrhythmia episodes recorded by CRT device, our study found that the LV dyssynchrony parameters as assessed by phase analysis of MPS were helpful for predicting the development of VT/VF in patients with ischemic cardiomyopathy after CRT. Further implantation of ICD in the device as CRT-D should especially be considered for those patients with bandwidth >139°.
